# Observations on Diabetes Mellitus

**Published:** 1886-09-20

**Authors:** William Bailey

**Affiliations:** Louisville; Professor of Materia Medica and Therapeutics, and Public Hygiene, in the Medical Department of the University of Louisville


### OBSERVATIONS ON DIABETES MELLITUS.

By William Bailey, A.M., M.D.,
**Professor of Materia Medica and Therapeutics, and Public Hygiene, in the Medical Department of the University of Louisville.**

As the name indicates this disease is especially characterized by the presence of sugar in the urine or glycosuria.

It is no wonder that for many years the disease was thought to be a disease of the kidneys, inasmuch as the chief manifestations of the disease concerned their functions.

This view was forever set at rest by Claude Bernard, whose experiments showed that glycosuria could be produced at will by puncturing, in the lower animals, the floor of the fourth ventricle of the brain, a space which since those observations has come to be regarded and styled the "diabetic area."

Before Bernard's experiments it was thought that diabetes mellitus was in some way dependent upon disease in the liver, especially after physiology determined its glycogenic functions.

By easy transition the seat of disease was transferred to the nervous centers, when it was established that artificial glycosuria could be produced at will by irritation of this diabetic area.

Even yet, both the etiology and pathology are in grave doubt, if not entirely undetermined.

This doubt will continue in force till the physiology of the subject is better developed. It seems apparent that the irritation of the diabetic area is reflected upon the liver so as to modify the amount and circulation of its blood, and in this way its function.

The blood in health contains more or less sugar for use in the economy, and it is stated that the kidneys will always eliminate it when the amount exceeds three parts to 1,000.

Sugar may no doubt be introduced directly into the blood by absorption from the alimentary canal without passing through the liver, depending of course upon what route it takes. This may be the case when the diet consists too largely of sugar or other carbo-hydrates.

I do not think that those cases in which sugar appears in the urine, evidently due to excess of sugar introduced into the system, should be classed as typical diabetes, mellitus.

These cases are not dependent upon any lesion of the nervous system; nor indeed is there any. The facts are that too much sugar by indiscretion has been introduced, and as the kidneys are responsible for the integrity of the blood. they simply remove it.

In the typical case, in my judgment, we have influence transmitted from the diabetic area to the liver, resulting in the passing of sugar in excess into the general circulation.

This effect is the same, whether the lesion be seated in this diabetic area at the base of the brain or whether some sensation is transmitted to this area, and then reflex influence sent to the liver, producing such changes in circulation and function that glyclosuria is developed.

The lesion need not be in this area, but may be situated anywhere, so that its nervous connection with it may result in this influence, being sent out to the liver from this center.

It has been shown that if you cut the pneumo-gastric nerve, and irritate the distal end that no glycosuria is produced; yet, if you irritate the proximal end of this cut nerve, you will send influence to the brain, which will be reflected upon the liver in such manner as to develop glycosuria.

Why may not the irritation of any distribution of the par vagus be taken up to center and reflected in this way?

Indeed we find glycosuria developed by impeded respiration, etc., where influences are sent up by the pneumo-gastric nerve. Many observers have found the disease in connection with organic disease in the pancreas.

Glycosuria is found under many circumstances, but it is not of grave import unless dependent upon involvement of the diabetic area either by lesion of the area itself or by lesion having nervous connection with it.

The disease is found most frequently in males between the ages of thirty and sixty years, yet it occurs in those much younger. Prognosis is rather more favorable in persons of forty and fifty years than in children.

Diagnosis is not often difficult, as the symptomology is very distinct and characteristic. The first indications are an increased frequency of micturition with a largely increased volume of urine of high specific gravity. Very soon great thirst is developed, with dryness and redness of the mucous membrane of the mouth, tongue, and fauces. The skin becomes dry, harsh, and deficient in secretion. Appetite may remain good or even be increased, and yet emaciation and debility rapidly progress.

The specific gravity of the urine may become very high notwithstanding the increased volume, usually 1,830 to 10,050, due to the sugar eliminated. The usual tests with copper will soon make the diagnosis complete. The increased function of the kidneys soon engenders changes in these organs. They become enlarged and take on changes according to circumstances. Complications are numerous, especially involving the lungs and skin.

Some form of phthisis or pneumonia, and in the skin boils, carbuncles, or some of the eruptive diseases.

Eczema is produced when the sugar comes in contact with the person. Duration of the disease varies from a few months to several years.

In addition to the above symptoms we have many others.

The temperature is usually below par, and when fever occurs from any cause it is not characterized by hyper-pyrexia.

Acetonæmia is sometimes developed with all of its train of symptoms, terminating in so-called diabetic coma.

Many disturbances of vision with various lesions of the eye also occur.

*Treatment.*—Treatment is usually divided into that that is medicinal, dietetic, and hygienic. Authorities place the greatest stress upon the dietetic, excluding from the dietary all articles containing sugar or anything easily converted into it.

An admirably arranged dietary may be found either in the excellent article on this subject found in the second volume of Pepper's System of Medicine, by Prof. Tyson; or in the paper read by Prof. Austin Flint, Jr., before the American Medical Association in 1884. These admirable papers furnish all the information desired on this point.

I do not think we shall ever be masters of the situation in the management of this disease until we are able to define and locate the essential lesion in each particular case, and by medicinal treatment eradicate the same; at the same time allowing the patient unrestricted diet.

No doubt, by dietetics, we may to a great extent remove the prominent symptom, glycosuria, from the case.

It is parallel to a restraint in a prison and unwilling abstinence from alcoholics for the cure of drunkeness.

The disease is cured only so long as the restraint is compulsory, the demon appetite continuing in force.

While I recognize that dietary is essential for the welfare of the patient, yet it is desirable that we should be able to remove the condition upon which the perverted function of the liver depends. Hence, I am inclined to place more stress upon the medicinal treatment.

Among the means of treatment I am inclined to place the use of codeia prominent. Next to, or perhaps equal to this, I shall place the bromide of arsenic, as introduced by Clemens and so fully appreciated by Prof. Flint, Jr.

Various remedies from time to time have been successfully used by many physicians in the management of this disease. Among the best we have ergot, the bromides, salicylates, etc.

The hygienic management is very important, and consists in surrounding the patient with conditions most favorable to health. Ventilated apartments, especially for sleeping; moderate exercise in the open air. Especial care should be taken of the skin by proper clothing, bathing and friction.

I have purposely avoided giving the minutiæ of the tests of sugar, the dietary, etc., as not being proper in a paper of this length. All these can be readily found in the papers already referred to, and also in many of the authorities.—*Progress.*

